# Bi-crystallographic lattice structure directs grain boundary motion under shear stress

**DOI:** 10.1038/srep13441

**Published:** 2015-08-25

**Authors:** Liang Wan, Weizhong Han, Kai Chen

**Affiliations:** 1 Center for Advancing Materials Performance from the Nanoscale, State Key Laboratory for Mechanical Behavior of Materials, Xi’an Jiaotong University, Xi’an 710049, China

## Abstract

Shear stress driven grain boundary (GB) migration was found to be a ubiquitous phenomenon in small grained polycrystalline materials. Here we show that the GB displacement shift complete (DSC) dislocation mechanism for GB shear coupled migration is still functioning even if the geometry orientation of the GBs deviates a few degrees from the appropriate coincidence site lattice (CSL) GBs. It means that any large angle GB can have a considerable chance to be such a “CSL-related GB” for which the shear coupled GB migration motion can happen by the GB DSC dislocation mechanism. We conclude that the CSL-DSC bi-crystallographic lattice structure in GB is the main reason that GB can migrate under shear stress.

Polycrystalline materials with grain sizes smaller than 1 μm can have strength much larger than their coarse grained counterparts due to the Hall-Petch strengthening effect^1^. In these small grained polycrystalline materials, grain boundaries (GBs) are an important ingredient of the microstructure that controls the mechanical behavior of the materials. Experiments show that GB-mediated plasticity (GB sliding, grain rotation, GB-dislocation reaction, etc.) is a major strain accommodation mechanism for the plastic deformation of these materials^1^. Meanwhile, rich experimental evidences demonstrate that grain growth can happen under various kinds of mechanical loadings for the small grained polycrystalline materials^2^,^3^,^4^,^5^,^6^,^7^,^8^,^9^,^10^,^11^. The grain growth in these circumstances has been proved to be largely attributed to the GB migration motion under shear stress^7^. Besides the grain growth, the shear stress induced GB migration behavior can also serve as a strain accommodation mechanism during mechanical loading of the materials^12^,^13^. Both effects should have profound influence on the mechanical performance of the small grained polycrystalline materials. However, the underlying mechanism for the GB migration under shear stress is still under debate.

Some geometric models of GBs like the dislocation model and disclination model have been proposed, and can seemly give explanation of this phenomena^12^,^13^,^14^,^15^,^16^,^17^,^18^,^19^. However, it is believed that any convincing mechanism for the shear stress induced GB migration behavior should take account of the atomistic structure of GBs and the motions of atoms in GB during its migration under shear stress. As concerning the structure of GBs, it has been pointed out many years ago that, there is a periodic bi-crystallographic lattice structure named as “coincidence site lattice (CSL)” for a group of GBs with particular misorientions of neighboring grains^20^,^21^. Moreover, a “displacement shift complete (DSC)” lattice which is the reciprocal lattice of the CSL can be defined for the CSL GBs^22^,^23^. The magic of the CSL-DSC lattice lies in the fact that, in a crystallographic sense, GBs can migrate by shear stress as long as the atoms in GB move according to a set of displacement vectors which connect the lattice sites of the neighboring grains in the GB zone correspondingly. As can be seen in Fig. 1b, the regularity in the displacement vectors makes that a GB DSC dislocation (or disconnection^24^,^25^ ), which is characterized by the Burgers vector ***b*** and step height *h*, can be defined^26^,^27^,^28^. The glide of GB DSC dislocation along the GB plane can then produce the slide-migration coupled motion of the GB^24^,^25^,^26^,^27^,^28^,^29^. The GB DSC dislocation mechanism is quite different from the migration of the GB by the uncoordinated atomic shuffling motion in the GB zone (see Fig. 1a). The random nature of the displacement of atoms in the latter case makes it unable to couple with any measurable transformation like the shear of the GBs.

The functioning of the GB DSC dislocation mechanism for GB shear coupled migration has been justified in previous studies for many low Σ CSL GBs with both symmetrical and asymmetrical tilt characters^30^,^31^,^32^,^33^,^34^,^35^. While it could be hard to make a definite map of all the CSL GBs that can give shear coupled migration by the GB DSC dislocation mechanism in specific materials, one can still establish that there should be a considerable amount of such GBs distributed in the 5-dimensional geometry space of GBs (misorientation ***θ*** = [*θ*_*x*_, *θ*_*y*_, *θ*_*z*_], inclination **φ** = [*φ*_*1*_, *φ*_*2*_])^22^. On the other hand, it has been proposed that, for GBs with misorientation or inclination deviates a few degrees from that of the exact CSL GBs (i.e., the so-called vicinal GBs), a GB secondary dislocation network can be introduced to accommodate the deviations in GB structure^36^,^37^,^38^. There are also a few studies demonstrate that some vicinal GBs can migrate under shear stress^38^,^39^. Nevertheless, it is not yet well known if the vicinal GBs in general can migrate under shear stress, and if the GB DSC dislocation mechanism still functions in shear of these vicinal GBs.

To address this issue, we performed atomistic modeling investigations on a series of vicinal Σ11 [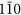] (1 1 3) and Σ9 [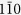] (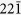) symmetric tilt GBs, and also the vicinal Σ3 [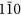]-tilt (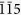)/(1 1 1) and Σ9 [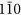]-tilt (1 1 5)/(1 1 1) asymmetric tilt GBs with an aluminum bicrystal model. Previous studies^32^,^33^,^35^ show that the structure of these CSL GBs in aluminum all agree well with the CSL theory description, and the GBs can migrate by shear of them along specific directions within the GB plane at room temperature. The kinematics of the shear coupled GB migration for these CSL GBs can be well explained by the GB DSC dislocation mechanism^32^,^33^,^35^. For the construction of the bicrystal models for these vicinal GBs, the lower grains of the reference CSL GBs alone were rotated by 4°, or both the neighboring grains of the reference CSL GBs were rotated separately by ±2°. The rotation axes were selected to be parallel or perpendicular to the GB planes of the corresponding CSL GBs. The particular rotation operation selected here is to make the vicinal GBs thus constructed be representative of a random vicinal GB in the polycrystals to a large extent. A summary of the geometric orientations of the vicinal GBs studied in this work can be found in the Supplementary Table S1. Molecular dynamics (MD) shear simulations were performed at 300 K for these vicinal GBs. Three different shear directions (X-, Y- axes of the simulation cell, and the angular bisector of the X-Y axes) within the GB plane were adopted for each of the vicinal GB.

## Results

As can be seen in the Fig. 2, these vicinal GBs can migrate upward or downward by shear of them along appropriate directions parallel to the GB plane (see also the Supplementary Table S1 and the Supplementary Movies). The stress-strain response for those shear simulations with shear directions aligned with the X- or Y- axes of the simulation cell has also been calculated, and the curves are given in the Supplementary Figure S2. For the study of the migration mechanisms, the structures of these vicinal GBs need to be analyzed first. Figure 3 gives some typical examples of the structure of the vicinal GBs studied. It shows that the structure of the vicinal GBs generally consists of areas of “good fit” intervened by GB secondary dislocations (marked by the red arrows and oval/rectangular frames in Fig. 3) distributed within the GB plane. Here, the area of “good fit” means that the arrangement of atoms in this area is almost the same as that of the reference CSL GBs. Take the vicinal GB “Σ11-113A” for example. As shown in Fig. 3a, the lower grain of this GB has been rotated around the [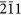] by 4° as compared with that of the Σ11 [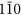] (1 1 3) symmetric tilt GB. The red arrows in Fig. 3a,b shows that a number of GB secondary dislocations aligned parallel to [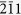] have been introduced to accommodate the deviations in misorientation and inclination. A step structure can also be formed by the GB secondary dislocations of this GB. On the other hand, the other areas in the GB plane have an atomistic structure the same as that of the Σ11 [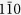] (1 1 3) symmetric tilt GB.

It should be noted that, the GB secondary dislocations shown here for the vicinal GB “Σ11-113A” generally have a compact core. However, for some other vicinal GBs, such as “Σ9-221A” and “AS-1-15/111A”, the areas as marked by the oval/rectangular frames in Fig. 3c–f show that the core of the GB secondary dislocations can be delocalized along the GB plane. Within the delocalized core of the GB secondary dislocations, the arrangement of atoms can have a minor disregistry as compared with that of the area of “good fit”. Nevertheless, it appears that the fundamental stacking pattern of atoms has been preserved to conform with that of the reference CSL GBs to a large extent. Given that the Σ11 [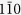] (1 1 3) symmetric tilt GB in aluminum has a relatively lower GB energy as compared with other CSL GBs^32^,^33^,^35^,^40^, one can infer that the delocalization of GB secondary dislocations is favored for the vicinal GB with a comparatively higher energy of its reference CSL GB.

With the particular GB structure as analyzed above for the vicinal GBs, it is reasonable to assume that the GB DSC dislocation mechanism of GB migration can be applied for shear of these GBs similar to their reference CSL GBs. To check this assumption, the ratio between the relative translation of neighboring grains and the GB migration distance for some of the shear processes were measured, and the results are given in Table 1. It should be mentioned that the relative translation of neighboring grains is actually a two dimensional vector parallel to the GB plane^33^. Here, we use the length of the vector in calculation of the ratio for simplicity. The ratio thus defined then conforms to the *β* factor usually used in literature^16^,^36^. On the other hand, a theoretical value of *β* (*β*^*theory*^) for the GB motions can be obtained by a careful analysis of the CSL-DSC lattice of the reference CSL GBs. The values of *β*^*theory*^ are also listed in Table 1 for comparison. A detailed analysis of how the values of *β*^*theory*^ were obtained here can be found in the Supplementary Material.

It can be seen from Table 1 that the measured values of *β* agree with the theoretical predictions quite well for some shear processes (Σ11-113B:X, Σ11-113B:X-Y, and Σ11-113C:X), but not good for the other ones. The agreement between the kinematic measurement and theoretical predictions indicates that the GB DSC dislocation can glide with very minor interference with the GB secondary dislocations for these shear processes. For the other shear processes, it may give that the GB secondary dislocations can interact with the GB DSC dislocations in a complicated way. The glide of GB DSC dislocations can be blocked by the GB secondary dislocations. In this case the relative in-plane translation between neighboring grains will be limited, as can be reflected by the smaller absolute values of *β* for the shear processes Σ11-113A:X and Σ11-113A:X-Y compared with the theoretical prediction (see Table 1). The arrows in Fig. 2a also indicate that point defects can be left over by the reaction of the preexisting GB secondary dislocations with the gliding GB DSC dislocations during shear of the vicinal GB “Σ11-113A”. Nevertheless, this does not mean the GB DSC dislocation mechanism fails, but indicates that some complications in the interaction between the GB DSC dislocation and GB secondary dislocations need to be considered.

On the other hand, it should be pointed out that there can be traps in the kinematic analysis of the GB motion with the aid of CSL-DSC lattice. The GB migration by the glide of GB DSC dislocations can sometimes give way to pure GB sliding^33^. Different GB DSC dislocations with different (***b**, h*) characteristics can sometimes function together one after another in shearing of the GB^33^. Moreover, there is no definite method that can determine all the possible GB DSC dislocations for a specific CSL GB. The method we used here is to simply look into the CSL-DSC lattice of the corresponding GB, and see which kind of GB DSC dislocations will likely to be functioning in practice as regard to the atoms displacements paths, the potential energy barriers, etc.^25^ As an example, the comparatively larger absolute values of the measured *β* to that of the theoretical predictions for the shear processes AS115/111A:X-Y and AS-1-15/111A:X as listed in Table 1 can be attributed to the fact that pure GB sliding has been involved during these shear processes.

## Discussion

**Figure 1:**
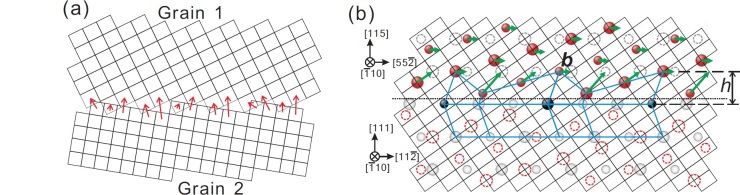
Atoms motions for GB migration. (**a**) Uncoordinated atomic shuffling motion (the red arrows) in the GB zone results in the GB migration. (**b**) GB migration by glide of a GB DSC dislocation (***b**, h*) on shear of the Σ9 [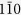]-tilt (1 1 5)/(1 1 1) asymmetric tilt GB in face centered cubic crystals. Red and light grey spheres represent the lattice sites of the upper and lower grains respectively. Red and grey dashed circles represent the extrapolating lattice sites of the upper and lower grains on the other side of the GB plane (marked by the dotted line) respectively. The black spheres are coincidence lattice sites. Spheres/circles of two different sizes correspond to the two alternating (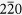) lattice planes. The fine grid is the DSC lattice.

The above results and analysis indicate that the vicinal GBs, which have misorientation and/or inclination close to that of the CSL GBs, can generally migrate under shear stress as well. The mechanism for the shear stress induced GB migration motion for these vicinal GBs can be rationalized as follows. With the deviation in misorientation and/or inclination as regard to the reference CSL GBs, a GB secondary dislocation network can be introduced to accommodate the geometry deviations. As can be seen in the Fig. 3, the introduction of the GB secondary dislocations also introduces a certain level of structure distortion of the GB in the core of the GB secondary dislocations as compared with the reference CSL GBs. This kind of structure distortion can serve as the block sites for the passage of the gliding GB DSC dislocations of the reference CSL GBs. However, the Fig. 3 also shows that the extent of this kind of structure distortion should be fairly small. This is especially true for the cases where the core of the GB secondary dislocations were delocalized (see Fig. 3c–f). One can thus imagine that the atoms in the core of the GB secondary dislocations can still find their way of motion under appropriate shear stress to allow the passage the GB DSC dislocations of the reference CSL GBs as illustrated in the Fig. 1b (see also the Supplementary Figures S3, S4, and S5).

**Figure 2:**
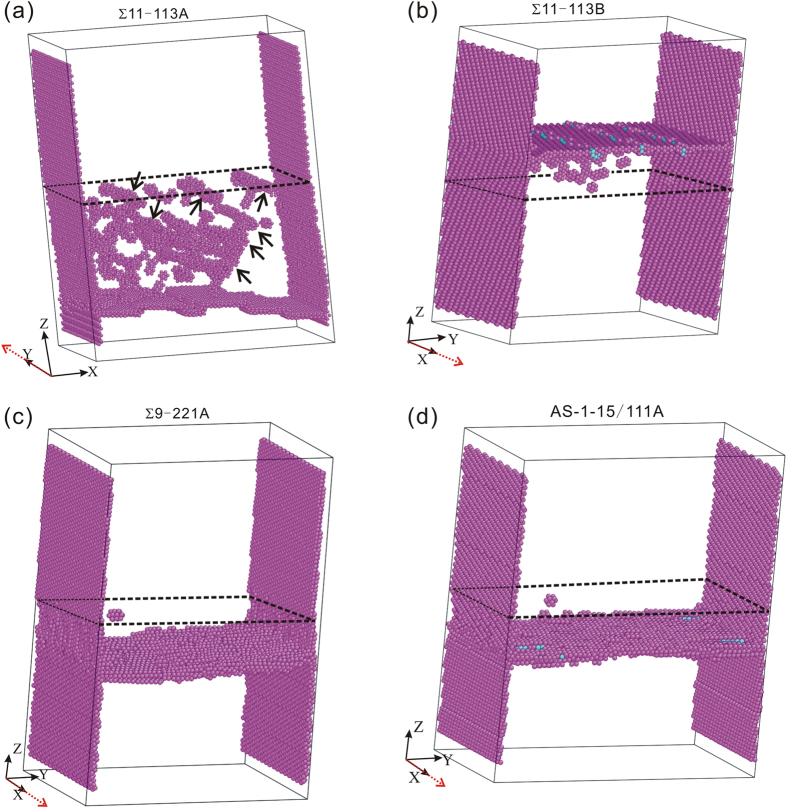
Snapshots of the final atomic configurations for shear of the bicrystals with the vicinal GBs. (**a**) Shear of the vicinal GB “Σ11-113A” along the Y-axis of the simulation cell. (**b**) Shear of the vicinal GB “Σ11-113B” along the X-axis of the simulation cell. (**c**) Shear of the vicinal GB “Σ9-221A” along the X-axis of the simulation cell. (**d**) Shear of the vicinal GB “AS-1-15/111A” along the X-axis of the simulation cell. Only the atoms which do not have the face centered cubic structural order (i.e., all the defects such as surfaces, GBs, point defects, etc.) are displayed. The shear directions are indicated by the red dotted arrows. The initial positions of the GBs are marked by the dashed frames.

**Figure 3:**
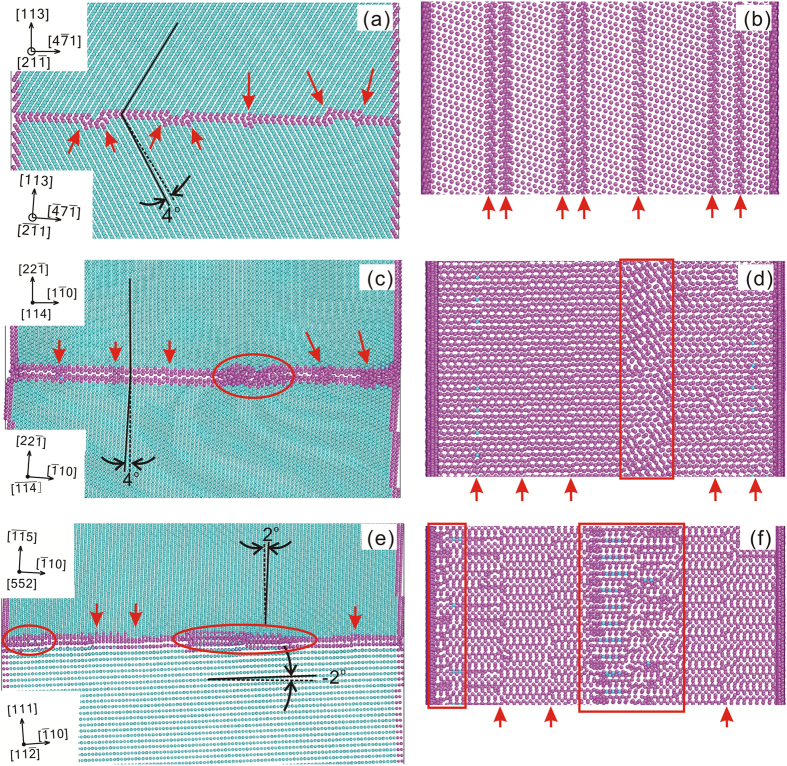
Structures of the vicinal GBs. Structures of the vicinal GBs “Σ11-113A” ((**a**) and (**b**)), “Σ9-221A” ((**c**) and (**d**)), and “AS-1-15/111A” ((**e**) and (**f**)). Atoms of face centered cubic, hexagonal close packed, and other structural orders are colored as turquoise, light blue, and pink respectively. The view direction of (**a**), (**c**) and (**e**) are parallel to the GB plane, while (**b**), (**d**) and (**f**) are the projection of the GB zone along the normal of the GB plane. In (**b**), (**d**) and (**f**), atoms of face centered cubic structural order are not displayed. The solid lines in (**a**), (**c**) and (**e**) mark the typical close packed lattice planes in the grains, while the dashed lines indicate the positions of the same close packed lattice planes in the reference CSL GBs. GB secondary dislocations are indicated by the red arrows or oval/rectangular frames.

The structure distortion in the core of the GB secondary dislocations can also lead to the uncoordinated atomic shuffling motions by the passage of the GB DSC dislocations. In this case, point defects as shown in the Fig. 2 can be formed and left over by the uncoordinated atomic shuffling motions there. This can be viewed as a result of the reaction between the gliding GB DSC dislocation and the preexisting GB secondary dislocations within the vicinal GB. It should be in some way similar to the point defect formation process by the dislocation dipole reaction during thermal annealing as found by atomistic modeling^41^. In a word, the GB DSC dislocation mechanism for the GB slide-migration coupled motion can still function even with the introduction of a certain amount of GB secondary dislocations for the vicinal GBs.

**Figure 4:**
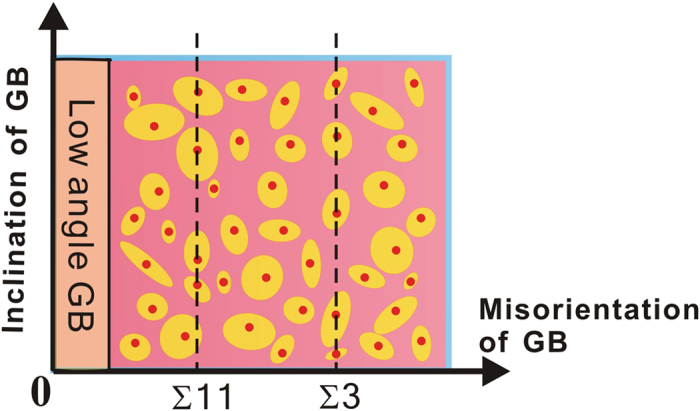
A GB diagram. A schematic diagram that illustrates the distribution of the “CSL-related” GBs (CSL GBs and their vicinal GBs) within the (misorientation ***θ*** = [*θ*_*x*_, *θ*_*y*_, *θ*_*z*_], inclination **φ** = [*φ*_*1*_, *φ*_*2*_]) five-dimensional space of the geometry of GBs. The CSL GBs that can give shear coupled migration by the GB DSC dislocation mechanism are represented by the red dots. The yellow shaded ovals surrounding the red dots stand for the corresponding vicinal GBs that can also produce shear coupled GB migration motion.

Take all the CSL GBs and the vicinal GBs that can migrate under shear stress into account, a class of “CSL-related” GBs can be defined as shown in the diagram of Fig. 4. It can be seen from this figure that, the “CSL-related” GBs which can give shear coupled migration take a quite measurable volume in the five-dimensional space of the geometry of GBs. It can thus be inferred that an arbitrarily misoriented and inclined large angle GB in small grained polycrystalline materials will have a considerable chance to be such a “CSL-related” GB. Although this study was performed with the aluminum which is a face centered cubic crystal, it is believed that the conclusion can be applied to other crystalline materials as well.

If we consider the stress-strain response of the shear processes obtained from the MD simulations (see Supplementary Figure S2) by taking account of the strain rate difference between the MD shear simulations and the usual mechanical experiments, one can roughly infer that the stress levels for the activation of the GB migration motion for these vicinal GBs are well within range of the stress condition of the experiments as reported in the literatures^32^,^42^. We thus emphasize that it is the bi-crystallographic lattice structure as formulated by the CSL-DSC lattice inherent to the GBs that underlies the shear coupled GB migration behavior as found widely in the experiments^2^,^3^,^4^,^5^,^6^,^7^,^8^,^9^,^10^,^11^. The shear stress driven GB migration behavior can have profound implications for the design of engineering polycrystalline materials with the GBs as the major structural defects.

## Methods

We used a bicrystal model (see Supplementary Fig. S1) with the X, Y, Z dimensions of the initial simulation cells of around 12 nm × 16 nm × 22 nm (about 220, 000 ~ 250, 000 atoms totally). For each vicinal GB, the orientations of the neighboring grains were determined as given in Supplementary Table S1. Due to the deviation in grain orientations to that of the reference CSL GBs, the lattice index of the X-, Y-, Z- planes in the rotated grains can be of irrational number. The periodicity of the rotated grains along the lateral dimensions (X, Y) can thus be broken. The free surface boundary condition was then used along the lateral dimensions (X, Y) of the bicrystal models when necessary. Two slabs along the Z-axis at the top and bottom of the simulation cell were designed to give the border region. The thickness of each border slab is around twice the cutoff distance of the interatomic potential for aluminum^43^ used in this work.

To obtain the room temperature equilibrium structure of GBs, an energy minimization procedure with standard conjugate gradient algorithm was performed on a set of initial trial GB structures. For all the GBs studied, 10 × 10 trial configurations of bicrystal with different in-plane rigid body relative translations of the neighboring grains were used to search for the optimized structure of the GBs. The 10 × 10 rigid body translation vectors were selected to give a uniform sampling of the cell of non-identical displacements (CNID)^36^ of the reference CSL GBs. The optimized structures obtained were then subjected to a MD annealing procedure to bring them to the equilibrium states at desired temperatures (300 K here) and zero stress.

The room temperature shear processes were simulated by the MD method. An integration time step of 2.0 fs was adopted throughout the MD simulations. The Melchionna modified Nose-Hoover dynamics^44^ was applied on the inner atoms between the border slabs for the control of the temperature and stress state of the bicrystals. For atoms belonging to the border slabs, the Z-axis component of their coordinates were scaled uniformly according to the length variation of the simulation cell in the Z-direction. Shear straining was applied by deforming the entire model as a whole. The shear directions were aligned with the X-axis, Y-axis, or the angular bisector of the X-Y axes of the simulation cell. For shear along the X-axis or Y-axis, the stress components in dimensions other than the shear direction were set to zero to allow the relaxation of bicrystals in these dimensions. A constant strain rate of 1 × 10^8^ s^−1^ was used for all the straining simulations. The stress tensor was calculated by the standard Virial expression^45^. For the measurement of the overall kinematics of GB motion, the external loading was first released after the shear straining processes.

All the simulations were performed with the LAMMPS code^46^. The visualization tool Atomeye^47^ was used to produce illustrations of defect structures of bicrystals. The common neighbor analysis (CNA) technique^48^ was used to give a classification of all the atoms according to their crystallinity (face centered cubic, hexagonal close packed, and other) in the visualization analysis.

## Additional Information

**How to cite this article**: Wan, L. *et al.* Bi-crystallographic lattice structure directs grain boundary motion under shear stress. *Sci. Rep.* **5**, 13441; doi: 10.1038/srep13441 (2015).

## Supplementary Material

Supplementary Information

Supplementary Information

Supplementary Information

Supplementary Information

Supplementary Information

Supplementary Information

Supplementary Information

Supplementary Information

## Figures and Tables

**Table 1:** The kinematics measurements of the GB motion for some of the shear processes. The shear processes are named by the ‘name of GB : shear direction’, see Supplementary Table S1.

**Shear processes**	***d***_***x***_	***d***_***y***_	***h***	***β***	***β***^***theory***^
Σ11-113A:Y	0.0	34.1	−81.3	−0.420	−1.225
Σ11-113A:X-Y	33.4	26.0	−50.3	−0.842	−1.225
Σ11-113B:X	38.5	0.0	29.4	1.307	1.225
Σ11-113B:X-Y	29.6	24.7	−31.8	−1.211	−1.225
Σ11-113C:X	33.3	0.0	24.3	1.372	1.225
AS115/111A:X-Y	24.1	20.1	21.2	1.480	0.707
AS-1-15/111A:X	35.1	0.0	−24.2	−1.450	−1.061

*d*_*x*_ and *d*_*y*_ are the two components of relative in-plane translation vector of neighboring grains along the X- and Y- axes respectively. *h* is the migration distance of GB with the positive (negative) value as upward (downward) migration. The unit of *d*_*x*_, *d*_*y*_ and *h* is Angstrom. *β* is calculated with 
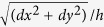
. *β*^*theory*^ is the theoretical value of *β* which is obtained by an analysis of the CSL-DSC lattice of the reference CSL GB (see Supplementary Material).
