# Supplementary material for: Bi-crystallographic lattice structure directs grain boundary motion under shear stress
Source: Sci Rep. 2015 Aug 25;5:13441. doi: 10.1038/srep13441 (PMC4548247; doi:10.1038/srep13441)
Supplement: Supplementary Information [file srep13441-s1.pdf]

Bi-crystallographic lattice structure directs grain boundary motion under shear stress

Liang Wan[1]*, Weizhong Han[1], Kai Chen[1]

[1]Center for Advancing Materials Performance from the Nanoscale, State Key Laboratory for Mechanical Behavior of Materials, Xi'an Jiaotong University, Xi'an 710049, China.

*Correspondence to: liangwan5@gmail.com.

Supplementary Materials include:

Supplementary Figure S1

Supplementary Table S1

Supplementary Figure S2

Supplementary Discussion with Supplementary Figures S3, S4, S5

Supplementary Movie Legends.

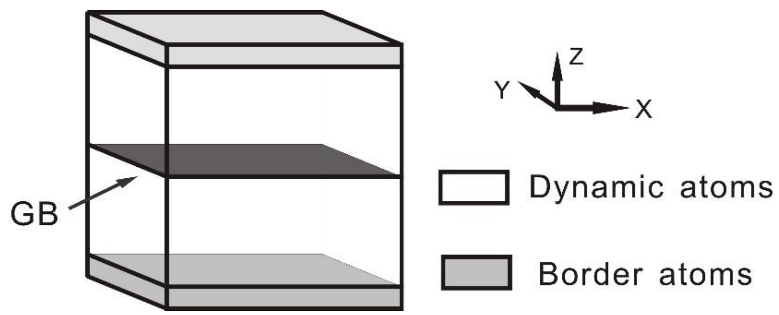

Supplementary Figure S1.

Geometry of the simulation cell and the bicrystal model used in this work.

Supplementary Table S1.

The bicrystal models and MD shear simulations performed for the vicinal GBs studied in this work. The spatial orientations of the reference CSL GBs are given by alignment of the X-, Y-, and Z- axes of the simulation cell with the particular lattice directions of the upper and lower grains of the CSL GBs. The vicinal GBs were obtained by a rotation of the lower grains alone or both grains of these reference CSL GBs around the X-, Y- or Z- axes by several degrees. In all the cases studied, the GB planes are perpendicular to the Z- axis. The shear directions were aligned with the X-axis, Y-axis, or the angular bisector of the X-Y axes of the simulation cell. The shear processes marked by [*] are the ones as presented in Table 1 in the article.

Name of vicinal GBs	Reference orientation		Rotation operation	Shear direction	GB motion
	Upper grain $(\boldsymbol{X\ Y\ Z})^T$	*Lower grain* $(\boldsymbol{X\ Y\ Z})^T$			
**Σ11-113A**	$[4\ \bar{7}\ 1]$<br>$[2\ 1\ \bar{1}]$<br>$[1\ 1\ 3]$	$[\bar{4}\ 7\ \bar{1}]$<br>$[\bar{2}\ \bar{1}\ 1]$<br>$[1\ 1\ 3]$	lower grain,<br>Y-axis, 4°	X<br>Y<br>X-Y	sliding<br>migration downward *<br>migration downward *
**Σ11-113B**	$[4\ \bar{7}\ 1]$<br>$[2\ 1\ \bar{1}]$<br>$[1\ 1\ 3]$	$[\bar{4}\ 7\ \bar{1}]$<br>$[\bar{2}\ \bar{1}\ 1]$<br>$[1\ 1\ 3]$	lower grain,<br>X-axis, 4°	X<br>Y<br>X-Y	migration upward *<br>migration downward<br>migration downward *
**Σ11-113C**	$[4\ \bar{7}\ 1]$<br>$[2\ 1\ \bar{1}]$<br>$[1\ 1\ 3]$	$[\bar{4}\ 7\ \bar{1}]$<br>$[\bar{2}\ \bar{1}\ 1]$<br>$[1\ 1\ 3]$	upper grain,<br>Z-axis, 2°<br>lower grain,<br>Z-axis, −2°	X<br>Y<br>X-Y	migration upward *<br>migration downward<br>migration downward
**Σ9-221A**	$[1\ 1\ 4]$<br>$[1\ \bar{1}\ 0]$<br>$[2\ 2\ \bar{1}]$	$[\bar{1}\ \bar{1}\ \bar{4}]$<br>$[\bar{1}\ 1\ 0]$<br>$[2\ 2\ \bar{1}]$	lower grain,<br>X-axis, 4°	X<br>Y<br>X-Y	migration downward<br>sliding<br>migration downward
**Σ9-221B**	$[1\ 1\ 4]$<br>$[1\ \bar{1}\ 0]$<br>$[2\ 2\ \bar{1}]$	$[\bar{1}\ \bar{1}\ \bar{4}]$<br>$[\bar{1}\ 1\ 0]$<br>$[2\ 2\ \bar{1}]$	upper grain,<br>X-axis, 2°<br>lower grain,<br>X-axis, −2°	X<br>Y<br>X-Y	migration downward<br>sliding + migration upward<br>sliding + migration downward
**AS115/111A**	$[5\ 5\ \bar{2}]$<br>$[\bar{1}\ 1\ 0]$<br>$[1\ 1\ 5]$	$[1\ 1\ \bar{2}]$<br>$[\bar{1}\ 1\ 0]$<br>$[1\ 1\ 1]$	upper grain,<br>X-axis, −2°<br>lower grain,<br>X-axis, 2°	X<br>Y<br>X-Y	sliding<br>sliding + migration downward<br>migration upward *
**AS115/111B**	$[5\ 5\ \bar{2}]$<br>$[\bar{1}\ 1\ 0]$<br>$[1\ 1\ 5]$	$[1\ 1\ \bar{2}]$<br>$[\bar{1}\ 1\ 0]$<br>$[1\ 1\ 1]$	upper grain,<br>Y-axis, 2°<br>lower grain,<br>Y-axis, −2°	X<br>Y<br>X-Y	splitting + migration<br>sliding<br>sliding + rotation
**AS-1-15/111A**	$[5\ 5\ 2]$<br>$[\bar{1}\ 1\ 0]$<br>$[\bar{1}\ \bar{1}\ 5]$	$[1\ 1\ \bar{2}]$<br>$[\bar{1}\ 1\ 0]$<br>$[1\ 1\ 1]$	upper grain,<br>X-axis, 2°<br>lower grain,<br>X-axis, −2°	X<br>Y<br>X-Y	migration downward *<br>migration downward + rotation<br>migration downward + rotation

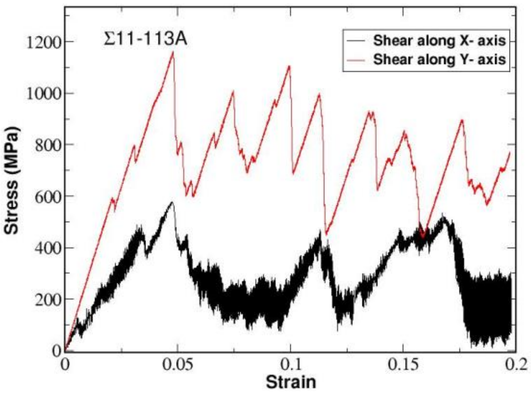
Σ11-113A
Shear along X- axis
Shear along Y- axis
Stress (MPa)
Strain
0
200
400
600
800
1000
1200
0.05
0.1
0.15
0.2

(a)

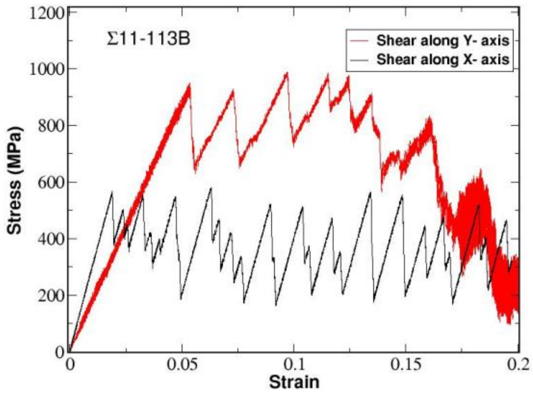
Σ11-113B
Shear along Y- axis
Shear along X- axis
Stress (MPa)
Strain
0
200
400
600
800
1000
1200
0.05
0.1
0.15
0.2

(b)

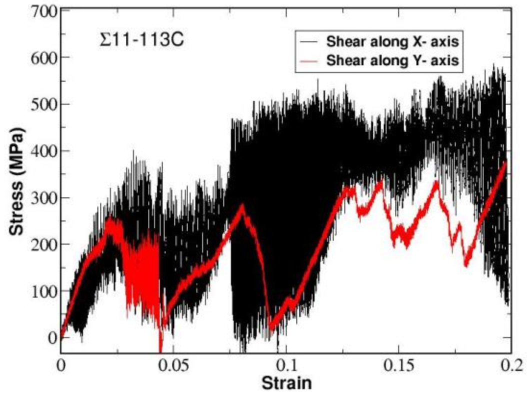
Σ11-113C
Shear along X- axis
Shear along Y- axis
Stress (MPa)
Strain
0
100
200
300
400
500
600
700
0.05
0.1
0.15
0.2

(c)

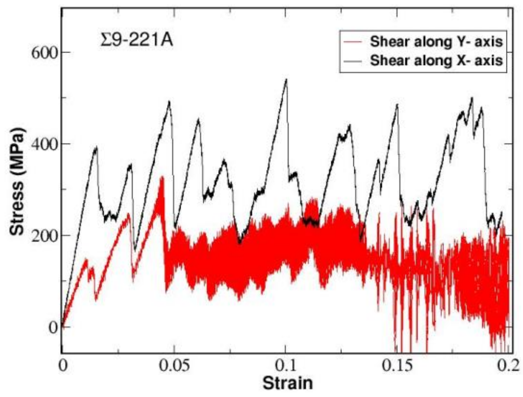
Σ9-221A
Shear along Y- axis
Shear along X- axis
Stress (MPa)
Strain
0
200
400
600
0.05
0.1
0.15
0.2

(d)

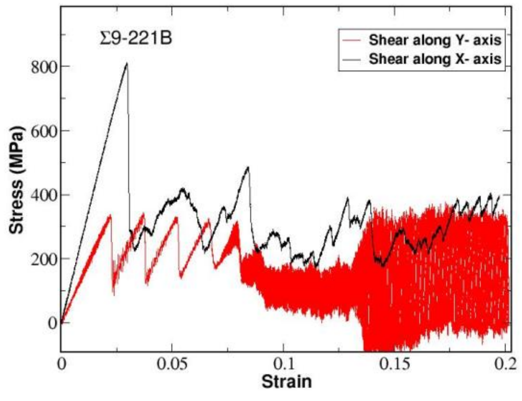
Σ9-221B
Shear along Y- axis
Shear along X- axis
Stress (MPa)
Strain
0
200
400
600
800
0.05
0.1
0.15
0.2

(e)

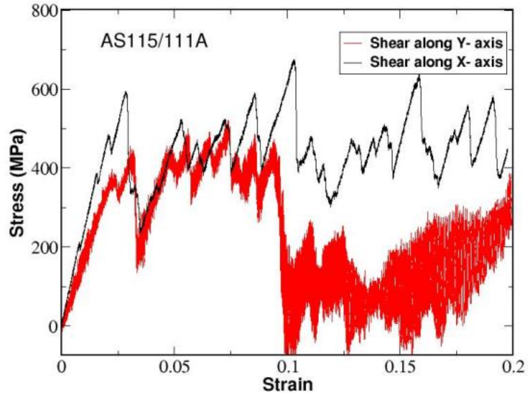
AS115/111A
Shear along Y- axis
Shear along X- axis
Stress (MPa)
Strain
0
200
400
600
800
0.05
0.1
0.15
0.2

(f)

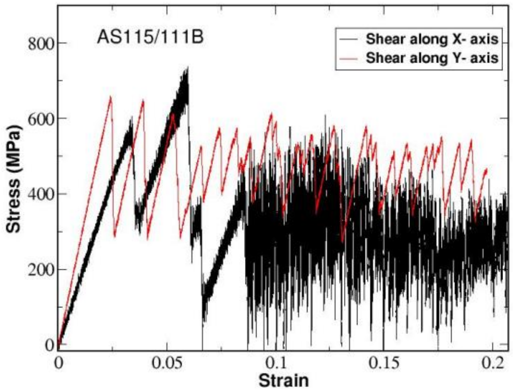

(**g**)

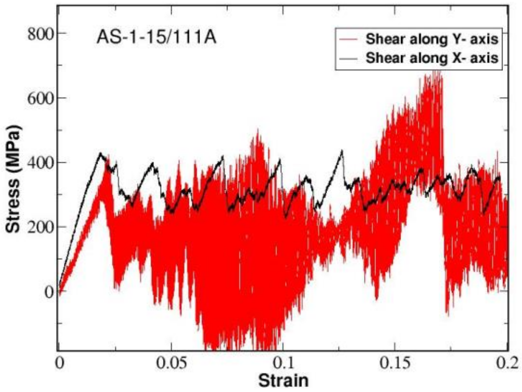

(**h**)

Supplementary Figure S2.

Stress-strain curves for the MD shear simulation of the vicinal GBs as listed in the supplementary Table S1. (**a**)-(**h**) correspond to the stress-strain curves for the MD shear simulation of the Σ11-113A, Σ11-113B, Σ11-113C, Σ9-221A, Σ9-221B, AS115/111A, AS115/111B, and AS-1-15/111A vicinal GBs respectively. In each panel, the black and red curves give the stress-strain response for shear of the GB along the X- and Y- axes of the simulation cell respectively.

Supplementary Discussion

Analysis of the theoretical values of β for the shear processes listed in Table 1

The theoretical value of β can be generally obtained by the $|\boldsymbol{b}|/h$ ratios of the GB DSC dislocations that function during the shear process. Based on a crystallographic justification and also an energy barrier estimation[1-4], the physically justifiable GB DSC dislocations and their ($\boldsymbol{b}$, h) characteristics can be figured out by a careful analysis of CSL-DSC lattice of the reference CSL GB for shear of the vicinal GBs. In the following, the CSL-DSC lattice analysis of the GB DSC dislocations for shear of the vicinal Σ11 $[1\bar{1}0]$ (1 1 3) symmetric tilt GBs, vicinal Σ3 $[1\bar{1}0]$-tilt $(\bar{1}\,\bar{1}\,5)$ / (1 1 1) asymmetric tilt GB, and vicinal Σ9 $[1\bar{1}0]$-tilt (1 1 5) / (1 1 1) asymmetric tilt GB as mentioned in Table 1 will be given. For a more comprehensive discussion of the GB DSC dislocation mechanism for shear of these reference CSL GBs, the readers are referred to the references 2 and 4.

1. Theoretical value of β for shear of the vicinal Σ11 $[1\bar{1}0]$ (1 1 3) symmetric tilt GBs

Figures S3a and S3b show the dichromatic pattern for the Σ11 $[1\bar{1}0]$ (1 1 3) symmetric tilt GB in face centered cubic crystals. By selecting the deep blue spheres as the upper grain and the light green spheres as the lower grain, the GB can be given as marked by the rhomb shaped dashed lines as show in the Fig. S3a. It can be seen that there are two possible GB DSC dislocations that can glide by shear of the GB: $\overrightarrow{AA}$ ($\boldsymbol{b}$ = (1/22) <4 7 1>, h = (1/11) |[1 1 3]|) and $\overrightarrow{BB}$ ($\boldsymbol{b}$ = (1/22) <3 3 2>, h = (2/11) |[1 1 3]|). With the particular shear directions as for the shear processes presented in Table 1, and consider that the length of Burgers vector and step height for $\overrightarrow{BB}$ are larger than that of the $\overrightarrow{AA}$, the GB DSC dislocation that functions in these shear processes should be $\overrightarrow{AA}$. The $|\boldsymbol{b}|/h$ ratio for $\overrightarrow{AA}$ can be calculated as (1/22) |<4 7 1>| / ((1/11) |[1 1 3]|) = 1.225. A negative value of −1.225 for the β^{theory} in Table 1 can be obtained by simply considering the symmetric counterpart of the GB DSC dislocation of $\overrightarrow{AA}$ in the CSL-DSC lattice here[2].

2. Theoretical value of β for shear of the vicinal Σ3 $[1\bar{1}0]$-tilt $(\bar{1}\bar{1}5)$ / (1 1 1) asymmetric tilt GB

Figures S4a and S4b show the dichromatic pattern for the Σ3 $[1\bar{1}0]$-tilt $(\bar{1}\bar{1}5)$ / (1 1 1) asymmetric tilt GB in face centered cubic crystals. By selecting the red spheres as the upper grain and the light grey spheres as the lower grain, the GB can be given as marked by the blue solid lines as shown in Fig. S4a. With a shear direction aligned with the $[\bar{5}\bar{5}2]$ of the upper grain as the case for the shear process of AS-1-15/111A:X in Table 1, atoms in the GB zone can move according to the green arrows shown in the Fig. S4. This corresponds to the glide of the GB DSC dislocation with $\boldsymbol{b}$ = (1/2) $[\bar{1}01]$ and h = - (1/3) |[1 1 1]| ($\overrightarrow{AB}$ in Fig. S4). On the other hand, the symmetric counterpart of this GB DSC dislocation as marked by the dash arrows in Fig. S4b has an equal chance to be activated by shear of the GB along this direction. A combined motion of both GB DSC dislocations will thus give a theoretical β value of (cos30°) • (1/2) $|[\bar{1}01]|$ / ((-1/3) |[1 1 1]|) = -1.061.

3. Theoretical value of β for shear of the vicinal Σ9 $[1\bar{1}0]$-tilt (1 1 5) / (1 1 1) asymmetric tilt GBs

Figures S5a and S5b show the dichromatic pattern for the Σ9 $[1\bar{1}0]$-tilt (1 1 5) / (1 1 1) asymmetric tilt GB in face centered cubic crystals. By selecting the red spheres as the upper grain and the light grey spheres as the lower grain, the GB can be given as marked by the blue solid lines as shown in Fig. S5a. With a shear direction aligned with the angular bisector of the X- and Y- axes of the simulation cell as the case for the shear process of AS115/111A:X-Y in Table 1, atoms in the GB zone can move according to the green arrows shown in the Fig. S5. This corresponds to the glide of the GB DSC dislocation with $\boldsymbol{b}$ = (1/6) $[2\bar{1}\bar{1}]$ and h = - (1/3) |[1 1 1]| ($\overrightarrow{CJ}$ in Fig. S5). It thus gives that β^{theory} = (1/6) $|[2\bar{1}\bar{1}]|$ / ((1/3) |[1 1 1]|) = 0.707 here.

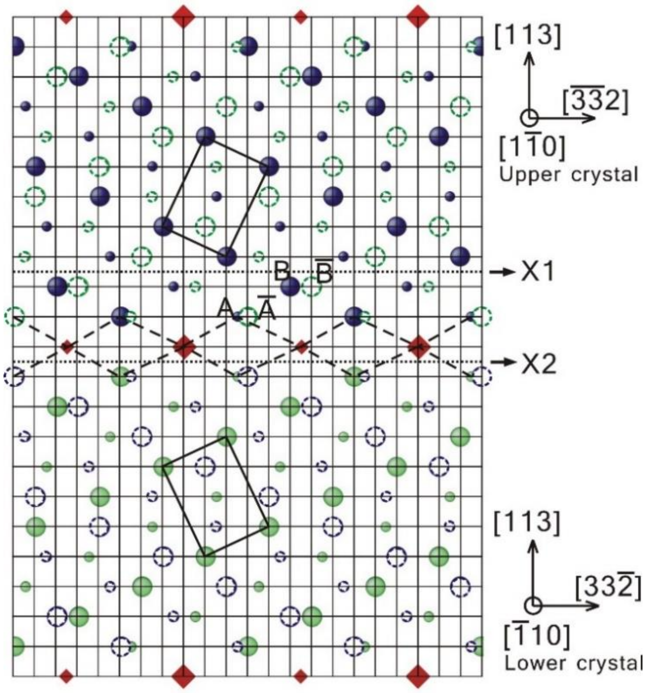

(**a**)

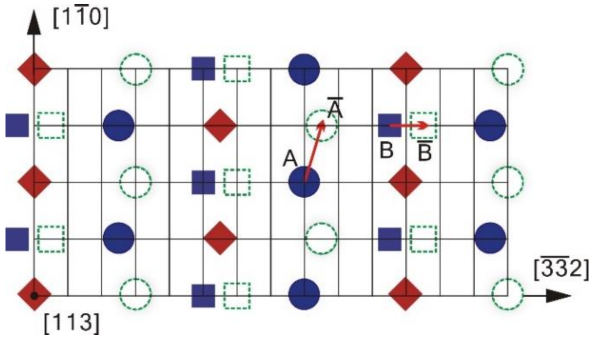

(**b**)

Supplementary Figure S3.

The dichromatic pattern for the Σ11 [$1\bar{1}0$] (1 1 3) symmetric tilt GB in face centered cubic crystals. (**a**) is the dichromatic pattern viewed along the tilt axis [$1\bar{1}0$]. Deep blue and light green spheres represent lattice sites of the upper and lower grains respectively. Deep blue and light green circles represent the extrapolating lattice sites of the upper and lower grains on the other side of the GB plane respectively. The red diamonds are the coincidence sites. The two different sizes of the spheres, circles or diamonds distinguish the two alternating ($2\bar{2}0$) lattice planes. The fine grid gives the DSC lattice. (**b**) is a projected view of the part of dichromatic pattern between the two cut planes X1 and X2 in (**a**) along the normal of the GB plane. The blank (or filled) circles, squares, and diamonds represent the three stacking lattice planes from the top to the bottom along the normal of the GB plane within this part of the dichromatic pattern. The color scheme is the same with that of (**a**).

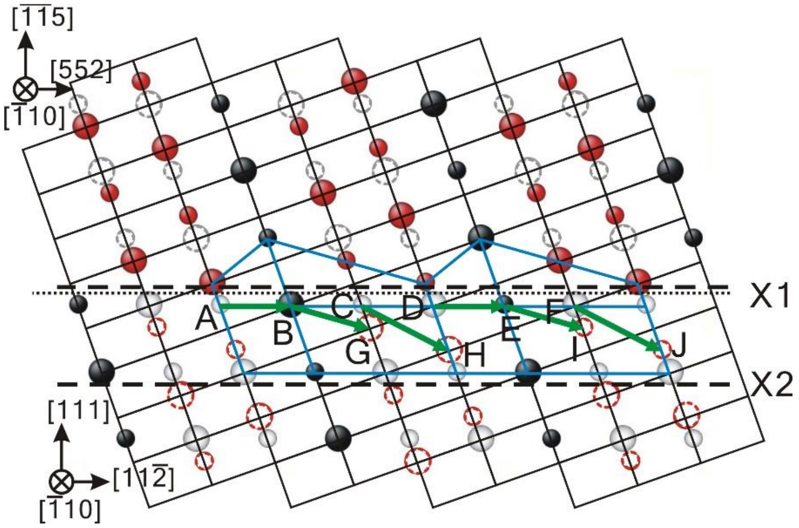

(**a**)

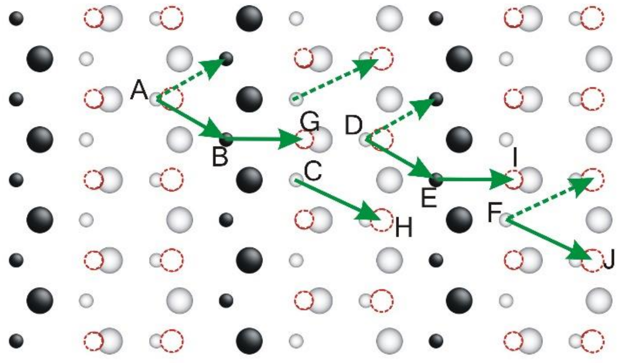

(**b**)

Supplementary Figure S4.

The dichromatic pattern for the Σ3 [$1\bar{1}0$]-tilt ($\bar{1}\,\bar{1}\,5$) / (1 1 1) asymmetric tilt GB in face centered cubic crystals. (**a**) is the dichromatic pattern of the GB viewed along the tilt axis. (**b**) is the projection of the part of lattice between the cut planes X1 and X2 marked in (**a**) viewed along the normal of the GB plane. Red and light grey spheres represent the lattice sites of the upper and lower grains respectively. Red and grey dashed circles represent the extrapolating lattice sites of the upper and lower grains on the other side of the GB plane (marked by the dotted line) respectively. The black spheres are coincidence sites. Spheres (or circles) of two different sizes in (**a**) correspond to the two alternating ($2\bar{2}0$) lattice planes. The fine grid is the DSC lattice. The size of spheres (or circles) in (**b**) is used to distinguish lattice sites of different heights along the GB normal, with the smaller spheres (or circles) lying higher than the larger ones.

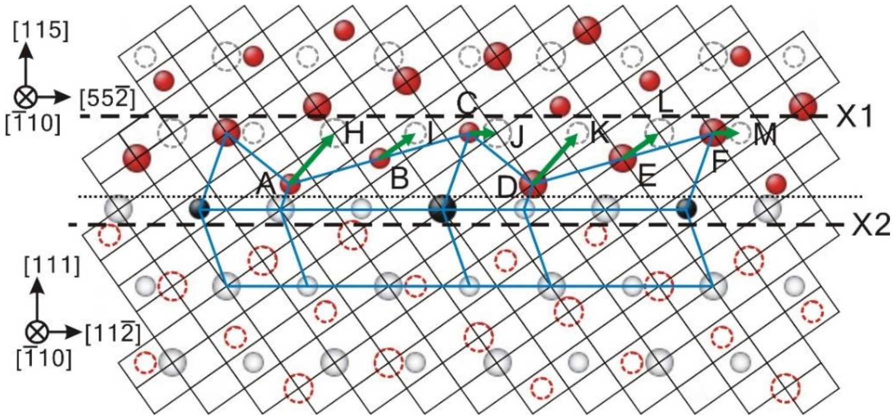

(**a**)

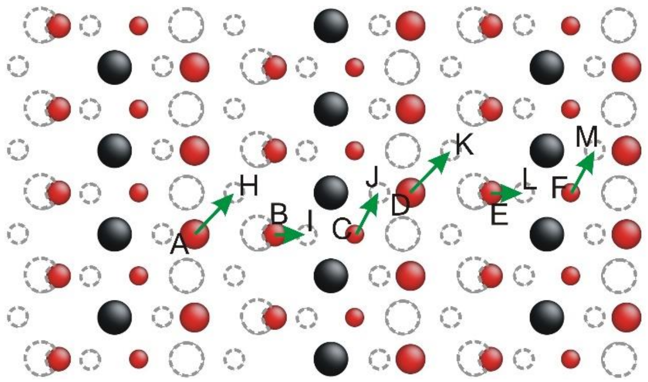

(**b**)

Supplementary Figure S5.

The dichromatic pattern for the Σ9 [$1\bar{1}0$]-tilt (1 1 5) / (1 1 1) asymmetric tilt GB in face centered cubic crystals. (**a**) is the dichromatic pattern of the GB viewed along the tilt axis. (**b**) is the projection of the part of lattice between the cut planes X1 and X2 marked in (**a**) viewed along the normal of the GB plane. Red and light grey spheres represent the lattice sites of the upper and lower grains respectively. Red and grey dashed circles represent the extrapolating lattice sites of the upper and lower grains on the other side of the GB plane (marked by the dotted line) respectively. The black spheres are coincidence sites. Spheres (or circles) of two different sizes in (**a**) correspond to the two alternating ($2\bar{2}0$) lattice planes. The fine grid is the DSC lattice. The size of spheres (or circles) in (**b**) is used to distinguish lattice sites of different heights along the GB normal, with the smaller spheres (or circles) lying higher than the larger ones.

Reference:

1 Pond, R. C. & Celotto, S. Special interfaces: military transformations. *International Materials Reviews* **48**, 225-245, doi:10.1179/095066003225010245 (2003).

2 Wan, L. & Wang, S. Shear response of the Σ11, ⟨1 1 0⟩{1 3 1} symmetric tilt grain boundary studied by molecular dynamics. *Modelling and Simulation in Materials Science and Engineering* **17**, 045008, doi:10.1088/0965-0393/17/4/045008 (2009).

3 Wan, L. & Wang, S. Shear response of the Σ9 ⟨110⟩ {221} symmetric tilt grain boundary in fcc metals studied by atomistic simulation methods. *Physical Review B* **82**, doi:10.1103/PhysRevB.82.214112 (2010).

4 Wan, L. & Li, J. Shear responses of [-110]-tilt {1 1 5}/{1 1 1} asymmetric tilt grain boundaries in fcc metals by atomistic simulations. *Modelling and Simulation in Materials Science and Engineering* **21**, 055013, doi:10.1088/0965-0393/21/5/055013 (2013).

Supplementary Movie Legends

Supplementary Movie 1. Animation of the MD simulated process for shear of the bicrystal with the vicinal GB 'Σ11-113A' along the Y- axis of the simulation cell.

Supplementary Movie 2. Animation of the MD simulated process for shear of the bicrystal with the vicinal GB 'Σ11-113A' along the angular bisector of the X-Y axes of the simulation cell.

Supplementary Movie 3. Animation of the MD simulated process for shear of the bicrystal with the vicinal GB 'Σ11-113B' along the X- axis of the simulation cell.

Supplementary Movie 4. Animation of the MD simulated process for shear of the bicrystal with the vicinal GB 'Σ11-113B' along the angular bisector of the X-Y axes of the simulation cell.

Supplementary Movie 5. Animation of the MD simulated process for shear of the bicrystal with the vicinal GB 'Σ11-113C' along the X- axis of the simulation cell.

Supplementary Movie 6. Animation of the MD simulated process for shear of the bicrystal with the vicinal GB 'AS115/111A' along the angular bisector of the X-Y axes of the simulation cell.

Supplementary Movie 7. Animation of the MD simulated process for shear of the bicrystal with the vicinal GB 'AS-1-15/111A' along the X- axis of the simulation cell.
